# Barriers and facilitators to the adoption of electronic clinical decision support systems: a qualitative interview study with UK general practitioners

**DOI:** 10.1186/s12911-021-01557-z

**Published:** 2021-06-21

**Authors:** Elizabeth Ford, Natalie Edelman, Laura Somers, Duncan Shrewsbury, Marcela Lopez Levy, Harm van Marwijk, Vasa Curcin, Talya Porat

**Affiliations:** 1 Department of Primary Care and Public Health, Brighton and Sussex Medical School, Watson Building, Village Way, Falmer, Brighton, BN1 9PH UK; 2 School of Sport and Health Sciences, University of Brighton, Brighton, UK; 3 Psychosocial Department, Centre for Researching and Embedding Human Rights (CREHR), Birkbeck College, London, UK; 4 School of Population Health and Environmental Sciences, King’s College London, London, UK; 5 Dyson School of Design Engineering, Imperial College London, London, UK

**Keywords:** Clinical decision support, General practice, Primary health care, Adoption, Barriers, Alert fatigue

## Abstract

**Background:**

Well-established electronic data capture in UK general practice means that algorithms, developed on patient data, can be used for automated clinical decision support systems (CDSSs). These can predict patient risk, help with prescribing safety, improve diagnosis and prompt clinicians to record extra data. However, there is persistent evidence of low uptake of CDSSs in the clinic. We interviewed UK General Practitioners (GPs) to understand what features of CDSSs, and the contexts of their use, facilitate or present barriers to their use.

**Methods:**

We interviewed 11 practicing GPs in London and South England using a semi-structured interview schedule and discussed a hypothetical CDSS that could detect early signs of dementia. We applied thematic analysis to the anonymised interview transcripts.

**Results:**

We identified three overarching themes: trust in individual CDSSs; usability of individual CDSSs; and usability of CDSSs in the broader practice context, to which nine subthemes contributed. Trust was affected by CDSS provenance, perceived threat to autonomy and clear management guidance. Usability was influenced by sensitivity to the patient context, CDSS flexibility, ease of control, and non-intrusiveness. CDSSs were more likely to be used by GPs if they did not contribute to alert proliferation and subsequent fatigue, or if GPs were provided with training in their use.

**Conclusions:**

Building on these findings we make a number of recommendations for CDSS developers to consider when bringing a new CDSS into GP patient records systems. These include co-producing CDSS with GPs to improve fit within clinic workflow and wider practice systems, ensuring a high level of accuracy and a clear clinical pathway, and providing CDSS training for practice staff. These recommendations may reduce the proliferation of unhelpful alerts that can result in important decision-support being ignored.

**Supplementary Information:**

The online version contains supplementary material available at 10.1186/s12911-021-01557-z.

## Introduction

The digital revolution has influenced many aspects of healthcare systems world-wide. United Kingdom (UK) based general practice (GP) has been at the forefront of this, with general practice patient notes moving in the 1990s from paper to Electronic Health Records (EHRs), using systems such as TPP SystmOne [1], EMIS Health [2], INPS Vision [3] and iSOFT [4]. Crucially, these are regulated and commissioned private companies that, like GPs, are contracted to deliver services to the NHS, but are not a core part of it, and thus have their own business models. Interoperability with the NHS is guaranteed by means of the GP IT Futures framework and the Accredited Services Register [5, 6]. This digitalisation means that all of the patient’s information is stored in one place, prescriptions can be sent to pharmacies electronically and many patients could access their healthcare records online at home if they so wished.

Data on patients’ demographics, clinical conditions and consultations are captured electronically, therefore they can be used to inform algorithms which could aid General Practitioners (GPs) in their consultations, risk assessments, diagnosis making, management choices, and safety netting [7, 8]. Known collectively as clinical decision support systems (CDSSs), these tools aim to perform a variety of functions and have different modes of delivery [9]. Some CDSSs exist as embedded templates that the GP can complete manually that come up without asking, while others are pre-filled, at the GPs’ request, with data previously recorded in the patient’s notes [10]. Some require activation by a clinician while others run automatically when a patient record is opened.

Most CDSSs perform one of the following functions: First, CDSSs can provide prescribing-based alerts (an inbuilt safety feature of EHRs). These pop-up messages remind prescribers to be cautious of prescribing medications to which the patient is known to be allergic, or prevent prescriptions being written if the patient is taking an interacting medication. Second, CDSSs can prompt clinicians to record important information in the patient’s EHR, in order to meet contractual requirements with the NHS (creating Summary Care Records, for instance), other set targets, such as the Quality and Outcomes Framework (QOF), or an electronic frailty index to assist the new frailty coding requirement [11]. QOF is a pay-for-performance scheme which aims to improve GP performance by offering financial rewards for practices who fulfil specific patient-centred criteria [12]. For example, practices are rewarded if their patients with diabetes have had appropriate reviews of glycaemic control within the last 12 months [13]. High quality data entry ensures records can be used to identify patients in need of a review for their condition or to create alerts on the patient’s home page to remind their GP to perform tasks such as measuring their blood pressure or glycated haemoglobin (HbA1C). Finally, CDSSs can indicate a patient’s risk of disease either currently (diagnostic) or in a pre-determined timescale (prognostic). For example, the QRISK score determines cardiovascular disease risk over 10 years, designed to prompt GP intervention to reduce risk [14].

CDSSs can aid the GP with the management of both individual patients and their overall caseload [15, 16]. However, there is considerable evidence of low uptake and dissatisfaction with use in General Practice settings, pointing overall to problems with individual CDSS purpose and delivery, and with how CDSSs interact with each other and the clinical context [17].

Many CDSSs remain un-implemented [18], despite extensive research into diagnostic CDSSs and the potential for these tools to improve patient outcomes [19]. The diagnosis gap for dementia in primary care provides one such exemplar case. Over 70 risk-prediction models for dementia have been developed [20] but none are currently used in UK general practice to aid GPs in picking up on patients at high risk of dementia, and around 34% of UK patients with dementia remain undiagnosed [21]. Although a dementia early detection or risk profiling tool may aid recognition of the condition in general practice, potential barriers to use of such tools include the likelihood of a high number of false positives which may cause harm to patients, and the lack of ameliorative treatments following diagnosis [22].

Many CDSSs also go unused because they are time-consuming for the GP to complete [23] and they see little additional benefit from completing them. While templates can collate information and provide useful explanations for patients [24], they may interrupt clinic workflow. The QOF scheme has been shown to improve patient outcomes for conditions like diabetes at a population level [25] but a particular patient may not wish to answer additional question considered less relevant for their current reason-for-the-encounter, and the scheme can be associated with the need for more GP consultations per patient, which puts additional stress on an already over-stretched service [26]. Although prescribing alerts are designed to improve patient safety, they are likely to be overridden if they are annoying, unhelpful, inefficient or irrelevant, or if the alert has not taken current patient context sufficiently into account [27, 28]. Further, due to the overwhelming presence of these, they are all too commonly dismissed without reading them [29], as the sheer volume of alerts in any given day can create the problem of alert fatigue [30]. Some of these issues above may explain why 49–96% of CDSS alerts are overridden or ignored [31].

Several commentators have described likely reasons for clinicians’ lack of engagement with CDSSs, suggesting that CDSSs have been developed with a poor match to clinical workflow, an excessive focus on interruptive notifications resulting in alert fatigue, and overly simplistic logic that fails to capture nuances of patient presentation or care [32, 33]. Primary care is basically reason-for-the- encounter led, which can easily be perceived to be at odds with a digital CDSS population-based approach in the 9 min available for a consultation. GPs may feel they would need to adapt their consultation style to make use of a CDSS [10]. This suggests that developers take an inadequate focus on processes of care, clinician workflow, or the whole EHR system, when developing CDSSs.

Confirming this, a review on artificial intelligence (AI) in primary care indicated that only 14% of published studies reporting development of AI diagnostic or treatment support algorithms had authors who were employed in primary care [34].

Together this evidence indicates that a variety of factors influence use of CDSSs, and that further contemporary qualitative research with UK GPs is warranted as a first step, particularly as the availability of CDSSs grow, their delivery evolves and a younger (and more fragmented) GP workforce emerges. In particular, low uptake and low perceived usefulness of CDSS by GPs indicates the need to align CDSS development to clinician needs and preferences, and the context in which they are delivered. This qualitative study was therefore conducted with the aim of supporting and optimising the design of future CDSSs by identifying factors that influence how or why GPs use these tools, looking specifically into aspects of CDSSs they find useful and problematic, both individually and in the wider context of their practice. The overarching aim was to ensure that CDSS design better accommodates and meets the needs of clinicians and the contexts in which they work.

## Methods

The reporting of this study follows the COnsolidated criteria for REporting Qualitative Studies (CORE-Q) [35].

### Participants and recruitment

Participants were recruited by convenience sampling through contacts at Brighton and Sussex Medical School (BSMS) and King’s College London Faculty of Life Sciences and Medicine. GP tutors or researchers at these medical schools were approached via email and in tutor planning meetings and invited to participate. To be eligible for the study the participants had to be currently working in general practice. Theoretical saturation of the data, whereby no further themes were identified, was reached after analysis of the 7th interview and verified by 4 more interviews; as such the sample size of 11 was deemed to be sufficient [36].

### Procedure

All interviews were conducted independently by a female freelance researcher (MLL) who holds a doctorate in Sociology, had extensive interviewing experience, and whom the participants had no relationship with, or knowledge of, prior to the interviews. Participants were interviewed by telephone or in a location convenient to them with reimbursement for any travel costs and for their time, no one else was present. The interview session began with the participants completing a demographics questionnaire followed by either signing a physical consent form or giving verbal consent if being interviewed by telephone. The interviews followed a semi-structured protocol using a topic guide, with questions such as “Are there any particular features or functions that you like in these types of computer systems providing advice, recommendations or alerts?” The topic guide is supplied as Additional file 1. This topic guide was pilot tested with one of the lead researchers (DS) and comprised a series of open-ended questions regarding the GPs’ views on CDSSs. At the beginning of the interview, a hypothetical example of a dementia early detection tool was introduced. This was chosen because there is a large body of work on developing automated dementia risk prediction work, some of which focuses on using primary care data [20, 37–42]. However, prediction or detection of dementia is still controversial because of a lack of treatment available which makes any different to the disease trajectory [43], because of the high risk of false positives [44], and due to the patient’s “right not to know” [45]. This context regarding a dementia-based CDSS was therefore explored throughout the interviews in order to give a focus and generate debate and discussion. All interviews were audio-recorded and transcribed verbatim by a third-party service (Essential Secretary Ltd); these transcripts were not returned to the participants. No repeat interviews were performed, and no field notes were recorded.

### Data analysis

We approached this analysis with a realist ontology and an objectivist epistemology. A thematic qualitative analysis was performed on the 11 interview transcripts by one coder (LS) using NVivo 12 [46]. The data analysis protocol followed the thematic analysis step-by-step guide provided by Braun and Clarke [47]. Firstly, the coder familiarised herself with the data by reading all transcripts thoroughly. The next step was to generate initial codes by highlighting parts of transcripts relevant to the study aims and tagging these to emerging codes. This was an iterative process, first coding a few transcripts, following which the codes were identified, named and defined, and a coding tree was produced, then the next few transcripts were analysed, to seek confirmation of initial codes and search for new ones. After 7 transcripts were analysed, no new codes or themes emerged. The coding structure was then evaluated by the study team (LS, EF, NE and TP) and codes were combined to form refined themes, which were derived from the data rather than specified in advance. The interview transcripts were then reanalysed looking for any further evidence of the themes identified. The study team once more discussed and refined the theme structure, to map it most closely onto the study aims. The participants were not asked to provide feedback on the findings from the data.

## Results

In total, 13 participants were interviewed, however, data from only 11 participants is included in this analysis as 1 GP refused to be recorded and 1 interview recording failed. The participants varied in age, role and other demographic factors as shown in Table 1 below. Regarding EHR use; 7 of the participants used EMIS Web, 3 used SystmOne and 1 was unknown. Interview time ranged from 22 to 53 min with a mean duration of 35 min.

**Table 1:** Participants characteristics

Characteristic	N
Age	
30–39 years	8
40–49 years	2
50 + years	1
Gender	
Male	7
Female	4
Ethnicity	
White British	7
British Asian	3
British other	1
GP role	
Partner	2
Salaried	7
Trainee	1
Hours worked	
Locum	1
Full time	2
Part time	9
Number of years practicing	
Median (years)	5
Range (years)	2–30
Location of practice	
London	5
Brighton	3
West Sussex	1
Kent	1
Somerset	1
Setting of practice	
Inner city	3
Urban	3
Suburban	2
Rural	3

Analysis of the 11 interviews led to the identification of nine themes organised across the following three over-arching themes:

- Trust in individual CDSS
- Usability of CDSS in consultation context
- Usability of CDSS in broader practice context

**Fig. 1:**
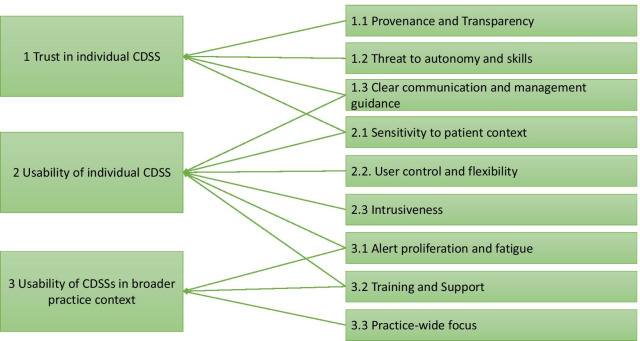
Coding tree detailing the themes and subthemes

Figure 1 illustrates how each of the nine themes relates to one or more of these over-arching themes, following which a description of each theme is given. Quotes are given that were determined to best illustrate the emergent theme.

### Overarching theme 1: trust in individual CDSSs

Trust can be defined as “a feeling of certainty that a person or thing will not fail”, [48] or as an “attitude that an agent will help achieve an individual’s goals in a situation characterised by uncertainty” [49], and this trust, or lack of it, mediates between people and their use of automation such as CDSSs. Trust in CDSSs can be developed by understanding what the origins of the technology and its outputs are, and learning over time whether the tool and its recommendations have a good fit to clinical workflow and context, and fit with clinicians’ self-view of their role in the clinic. These facets of promoting trust in CDSS are described below.

#### Provenance and transparency

The first theme affecting GPs’ trust in CDSS was the tools’ provenance, i.e. its origins and the means by which it was developed; together with how transparently this information was available. Poor uptake was associated with a lack of knowledge about the research underpinning its development:

“They're inherently distrustful of anything that hasn’t got somebody saying ‘oh I've done this trial and this trial and we've used it’ and I think that's what we've been taught at medical school; to be sceptical until we've got the evidence.” – P06

Some participants suggested GPs were more likely to pay attention to an alert generated by a member of staff than an automated computer-generated alert:

“I think it's just that you, you feel like actually someone’s thought about that, you know, and it's a clinician who's thought about it rather than it just being, you know, automatically raised and may not actually be relevant to the patient.” – P04

CDSSs that other GPs or practices had found useful and had recommended were more likely to be trusted and used. This suggests the importance of the tool aligning to clinical workflow and of GP involvement in tool design and dissemination, as if another GP has found it useful and endorsed it, a new GP is more likely to be ready to try it:

“I think there is a degree of sharing these kind of protocols and algorithms amongst GP practises and we certainly, you know, GPs talk to each other and say ‘oh yeah we've got this thing that alerts for that particular problem’, ‘oh, have you? Right great can we, can we bring it in?’.” – P06

#### Threat to autonomy and skills

Participants’ feelings of distrust in CDSS reflected a concern that such tools represented a threat to GPs’ knowledge and autonomy by ‘questioning’ the accuracy and decision-making skills of practitioners:

*“I think we don’t like using it because we think we’re better than computers.” – P05*.

However, some GPs acknowledged that the CDSS could add value to their practice:

“…there is an element of arrogance to that which is saying that we know better than all of these algorithms and things...” – P06

CDSSs were perceived as useful and less threatening where they acted as an aid-memoire to ensure all aspects of assessment were incorporated:

“I use a menopause one. It will … remind you of everything you should be asking in that consultation, so it’s a little bit like consultations for monkeys I suppose, but really a help, actually a helpful aide-memoire.” - P05

“We really do like those prompts in a patient with diabetes where there are 17 things to do and I’ve just missed out two or three of them, so it’s really nice to have somebody say ‘oh don’t forget there’s the urine test and you haven’t yet sent the patient for the eye test that you thought somebody else was doing and it looks like nobody’s done after all, so maybe get that done’.” – P02

Alerts were particularly welcomed, and less likely to be perceived as a threat, when they were developed as a result of requests by GPs to improve practice, rather than imposed on GPs from an unknown or non-clinical source:

“The GPs themselves were asking for an alert, and the alert was, in pregnancy, second, third trimester to give the pertussis vaccine and because the GPs were asking for that prompt and wanted to be reminded and didn’t want anybody to slip through the net, they all took really good notice of it.” – P02

#### Clear communication and management guidance

This theme related to two over-arching themes—Trust in individual CDSS and Usability of individual CDSS. One of the most frequently discussed topics was the importance of the CDSS outcome being simple to explain to patients. A frequently-given example was the QRISK pictogram demonstrating the patient’s risk in an easy-to-understand form that explains the necessity of treatment:

“It actually comes up with a nice diagram that it can use with your patients that’s got smiley faces of different colours to illustrate risk … it’s just a different way to help augment that communication with them. For things like that it’s quite helpful because patients are wary of tablets, quite rightly. They don’t quite understand ‘oh, what does it mean that I’ve got a 20% risk of, of an event within the next 10 years’, you’re like, ‘well actually in a crowd of people if we were to revisit them in 10 years’ time two of them would have had a heart attack’ or something like that, it, it’s just helpful.” – P03

Having a clear and easy-to-understand outcome was not only a feature that is beneficial to patients, it could be important to the GP. Tools that converted inputted data into an easy-to-follow management plan (e.g. the ‘Sick Child Template’) were perceived as useful:

“It's got a kind of column of green things, a column of orange things and a column of red things. Then there's a really clear next page about what you should do if they're kind of, if they’ve got lots of greens, you know, what the process would be if they’ve got lots of reds in terms of, you know, one side and I think that’s really helpful just because it kind of combines the data you're putting in with actually a useful plan.” – P04

### Overarching theme 2: usability of individual CDSS

Usability is ‘the extent to which a product can be used by specified users to achieve specified goals with effectiveness, efficiency, and satisfaction in a specified context of use’ [50]. Usability was influenced by whether the CDSS effectively supported the decisions of the GP, or conversely if the CDSS was ignorant to the patient context and therefore inaccurate. CDSSs were perceived as efficient if they supported the GP’s decision-making in a non-intrusive and flexible way, and user satisfaction could be understood as whether the GP found the CDSS as a good fit to their workflow, or conversely found it frustrating. These facets of usability are explored in the three sub-themes described below.

#### Sensitivity to patient context

Although under overarching theme 2, this theme also relates to Trust in Individual CDSS and encompasses both the accuracy and appropriateness of the CDSS to give useful decisions. The additional contextual information that a GP, but not a CDSS, is privy to, will affect the accuracy of the tool output. This led GPs rightly to question the sensitivity and specificity of individual CDSS, as illustrated in this example:

“I had a patient…who scored very poorly in the … cook-book scoring system [for diagnosing memory problems]. If she was doing that online she'd have been sent off to a memory clinic but actually she was severely dyslexic… she's never been good with being able to tell the time.” – P10

Also mentioned was the GP’s familiarity with the patient and the benefit of observation during face to face visits:

“A lot of the patients who I kind of see regularly with chronic problems, I kind of know if they're well or not because I know the patient and I just can tell if they're themselves or if they're not because you have a constant, you know, you lose that with a computer.” – P04

“… I can tell… in the three-metre walk from the waiting room to my consultation room whether I'm going to send the child into hospital or not because I can just see if they look well or not.” – P04

Additionally, GPs discussed problems with not only CDSS accuracy but with appropriateness in the broader clinical care of each patient. Once again, participants described how the computer cannot take account of social cues or the wider patient context within the consultation. One participant raised this in relation to dementia diagnosis:

“Actually, if that patient is, you know, probably in the last six months of their life, or they're falling over all the time, or they're getting really confused with their medications, it might be completely inappropriate and then you’ve got just a whole world of paperwork and alerts that actually aren't relevant because it's the computer system that’s making you do it.” – P04

Participants also noted the negative physical and emotional impacts on the patient of unnecessary investigation:

“The problem comes in where….it leads to over-investigation of a patient” – P09

“Now, how is a patient going to feel if I’m doing their diabetes check and then up pops ‘this patient may have dementia … please test’, then that’s immediately going to get them pretty worried” – P02

Similarly, one respondent noted that both screening and diagnosis are only important to carry out where the patient will benefit as a consequence; this was particularly mentioned in relation to dementia screening:

“The worst thing you can do is to have an earlier diagnosis but there’s no services anywhere that, there’s no psycho-geriatric elderly mental health care services to refer the patient into, so you’re just suddenly creating this demand with no potential outlet and that really increases frustration.” – P02

#### User control and flexibility

Usability was also improved by flexibility in the presentation of the CDSS, e.g. the option to dismiss it or leave certain fields incomplete. GPs explained that this allowed them to be in control of CDSSs rather than feel controlled by them. This came up firstly in relation to templates requiring information irrelevant to a particular consultation:

“If it’s optional it’s not a problem. I think there have been templates designed before that you have to enter everything and that’s very frustrating.” - P01

“I think it feels frustrating … when the system is so process-driven that there's no sort of autonomy of the GP to, you know, change it or, kind of, put in what’s relevant.” – P04

Similarly, GPs expressed frustration at being unable to alter the outcome of some CDSSs:

“You can click ‘escalation not appropriate’, but then it automatically codes sepsis or something in the notes and you can’t do anything about that.” - P08

Frustration was also expressed that some CDSSs required description of actions taken after consultation, using inflexible options, leaving GPs the choice to record falsehoods or leave an audit trail in which they felt they might be culpable:

“… in order to dismiss it you have to select what action you’ve taken such as, well have you called an ambulance, have you sent them into hospital…you’re then panicking thinking gosh I don’t want, I don’t want to lie, no I haven’t sent them into hospital but is that a judgement, is that them saying because I haven’t done that I’m a rubbish doctor?”- P03

Generally, CDSS flexibility and control was valued as a means of maintaining GP autonomy, linking ability to control the functions of the CDSS with control of making the best decisions for individual patients:

“And I think what the clincher is…it’s seen to be something that the GP is leading or … is it imposed upon the GPs... The computer is there to support the role of the doctor and not in any way to takeover.” – P02

#### Intrusiveness

The intrusiveness of CDSSs was perceived variously as helpful and unhelpful. Two participants particularly highlighted the potential usefulness of dementia screening tools due to difficulties in assessment and early diagnosis:

“Something like dementia you do want to identify it early, and actually in terms of dementia I think it would be helpful to have a decision aid because at the moment it’s, it’s not very easy to assess patients for dementia.” – P01

“… making the diagnosis actually can be really helpful because it gives a kind of rationale and it gives a reason for, you know, the symptoms the person might have, and it can give access to support if then you've got a diagnosis of dementia” – P04

Conversely, CDSSs were perceived as an unwelcome intrusion where they raised an issue which the GP felt to be unimportant within that particular consultation. The “agenda” of the consultation may be determined by the patient or the GP, but where an alert presents which does not relate to a topic of importance for either party in the consultation, it may be perceived as undermining of the GP’s professional expertise and would be unlikely to be used:

“So, if it seems to address the GP’s own agenda then maybe they’ll take notice, if it doesn’t then they’ll find them deeply de-professionalising, and concentrate on the patient in front of them and not the computer screen in front of them.” - P02

Given the time-pressured environment that the GPs work in, self-population of CDSS fields using previously-recorded data (e.g. in QRISK) was viewed as a benefit which reduced intrusiveness:

“So, by the time you open the tool, you might only have to … click a couple of boxes and then you're done.” – P09

Conversely, poor integration of individual CDSSs into the computer system created difficulty when data was requested that could not be accessed without closing the CDSS template:

“You can’t go looking in the notes, you can’t input anything else, this is now taking priority, so you either suspend it and then re-open it and suspend it and re-open it, which is just a hassle.” – P05

### Overarching theme 3: usability of CDSSs in the broader practice context

This overarching usability theme considered CDSSs beyond their individual features. Each CDSS exists in the context of multiple competing CDSSs and other alerts, templates and forms, that can influence their uptake and use.

#### Alert proliferation and fatigue

Intrusive alerts were frustrating on their own, but in the context of the proliferation of multiple intrusions and alerts, participants described experiencing “alert fatigue”. This was the cumulative and distracting effect of multiple competing demands coming from the computer system:

“In general practice you might have the receptionist sending you an instant message, you might have a notification coming from the practice team, you might have a task coming through that a prescription needs to be done, so you might have four or five pop ups anyway.” – P05

In particular, their distracting effect was seen to negatively affect patient-doctor rapport:

“It really is like an interruption, you know, no GP wants somebody to just burst in with the door opening or the phone ringing. In the same vein no GP really wants a big thing to just pop up on the screen that they didn’t call up.” – P01

“And then the patient is talking to you and I have had to say… ‘I’m really sorry but I was distracted by the messages popped up on my screen and can you please tell me about it again’.” – P05

Having to handle the constant distractions from multiple sources meant the GPs had to attempt to multitask, which they considered to present a patient safety risk:

“So, you’re confronted with that need to sort of multi-task which isn’t safe… I think most people can’t really safely multi-task especially when you’ve got actual decisions that you’ve got to make.” – P03

The proliferation of alerts led to participants becoming desensitised to alerts, with GPs overriding or dismissing them:

“You get into a habit and it, it’s something that we all joke about professionally, you get into a habit of just dismiss, dismiss and click on the X, or find some way, you don’t even read it properly now.” – P03

“Because I think we get to the point where we get so bombarded and overwhelmed with stuff that we just start to switch off from these computer alerts, you know”– P10

This fatigue could also cause GPs to miss important alerts, with ramifications for patient safety:

“You end up getting so many alerts that it's very difficult to see the woods for the trees with them.” – P06

“I prescribed Trimethoprim for somebody and didn’t notice an alert, I must have dismissed it, that said they were allergic to Trimethoprim, and it was only the pharmacist calling up saying did you realise, you didn’t mean that did you?” – P03

#### Training and support

Training and support emerged as a key theme related to both usability of individual CDSSs and their general use in the overall practice context. Firstly, training needs were perceived to differ by age and to be related to overall confidence with information technology:

“I think to a certain extent there is a bit of a cultural change in the sense that the newer generation of doctors coming through are much more used to computers telling them things in their own lives and so they're much more accepting of the idea that the computer might give them helpful information.”- P06

GPs often felt irritated that new tools or system updates were added but with no explanation of how best to use them:

“I suppose my, my biggest bug bear is training, if anything new comes in it then needs to be disseminated to everybody who’s using the system, which often doesn’t happen.” – P05

Related to this, GPs described that many CDSSs were incorporated into EHRs but could get ‘lost’ amongst other system features without adequate training:

“You know and that's how I enter it into the notes that I've, that I've calculated it rather than using the internal scoring system which EMIS ... probably has, I just haven't found it yet.”- P10

Familiarity with not just information technology overall, but the individual tool itself was seen to improve uptake:

“I think if you’re very familiar with a, with a template then you can probably use it all the time and it probably does speed things up”- P07

#### Practice-wide focus

CDSS use which operated at the broader practice level was seen as a helpful endeavour when still controlled by the practice or GP, in order to generate lists of possible missed diagnoses or patients who should be screened for particular conditions:

“I think there's some people who really clearly fall into the category you know even sort of middle ones, but sometimes people do get missed… I think sometimes having an automated system plus the option for GPs whether to add patients to that list.” – P04

“Overall, I think GPs have an important role in improving public and population health, and even if we did, say, okay, these 5% of patients may be at risk of dementia, we need to do this work and call them in and try to diagnose the dementia earlier, that will be better for the population’s health going forward.” – P01

Practice control of list generation (as opposed to automatically-generated CDSSs popping-up during consultation) was seen as an important means of keeping GP workloads at manageable levels:

“That would just mean that as a GP you’ve then got to kind of go and do, you know, a mental state, cognitive state, exam on every patient who says they might have dementia which would just kind of create workload and when are you going to do that?” – P04

## Discussion

We have identified features of CDSSs, individually and in the broader practice context, which promote or hinder their uptake by GPs in the UK. Our results suggest that many previous CDSSs have not been designed with the end user, practice context or clinical workflow at the forefront, and thus remain difficult to use. Commercial interests may also play a role here due to the NHS purchase/provider split. There is thus substantial room for improvement in the development and implementation of these tools. First of all, results suggest it is important that the provenance of the CDSSs is congruent with sources which are esteemed as trustworthy or scientifically credible by the GP and that this provenance should be easily accessible to GPs. Similarly, CDSSs’ accuracy was valued, particularly when identified through robust validation studies, which had taken account of the broader patient context. GPs perceived CDSSs to be most useful when they provided clear guidance on actions to be taken following use; this could take the form of a clinical guideline or pathway, or a communication aid for discussing next options with the patient.

Participants described that CDSSs needed to be technically well integrated into their patient record systems, and be designed to both reduce manual information entry (e.g. by harvesting it automatically from the patient record) and be flexible enough to be dismissed when the pop-up was out of context. CDSSs which needed to be manually filled in, but which blocked out access to the patient record while open, were especially difficult to use and disliked by GPs.

Practitioners described wanting CDSSs to assist, rather than dictate, their clinical practice, so as not to be a threat to their autonomy. GPs did not want to feel de-professionalised by the computer over-riding their decisions or intruding where it was not appropriate; where this happens, it may indicate that tools are poorly developed and tested. It was observed that GPs could have complex views on CDSSs, as pop-up reminders and guidance on decision-making were experienced as both helpful and problematic, depending on the context of the individual consultation (how the patient presented, and the competing agendas of patient, clinician and CDSS), who initiated the request for the CDSS, and on the degree of proliferation. These perhaps in part reflect – and are solved by – integration of CDSSs and practice-level training, the latter also providing an opportunity to present the tool’s provenance and evidence for its accuracy. Many GPs indicated that CDSSs appeared in their systems with no training or support on how to use them, which meant that they only learned how to disable them, rather than engage with them fully and utilise them to their best potential. Results also indicate the need to involve GPs and other practice staff in CDSSs design, in order to prevent CDSSs being perceived as a threat, but rather to be seen as a tool complementing practitioner’s knowledge and autonomy.

The GPs in our study acknowledged that screening and risk profiling tools could help to pick up cases of important diseases such as dementia, but were wary of over-diagnosis, over-investigation of ultimately well patients or diagnosis and screening inappropriate in the context of a given patient’s co-morbidities or other life challenges. They viewed this excessive intervention as a potential source of patient harm. This intersects with the need for high accuracy in tools and also flexibility to decline tool use, for example in the given example of a patient in the end-stages of life. GPs felt that CDSSs which could compile concise patient lists of people who warranted further investigation for diagnosis or preventative care might be helpful in the wider practice context.

### Findings in the broader research context

Previous work investigating clinicians’ adoption or non-adoption of CDSSs has shown a range of similar problems causing low uptake of tools in the clinic, such as templates being time-consuming to complete [23], poor training on using alerts [9, 51, 52] alerts being over-ridden [29, 31] or irrelevant [28] and clinicians being fatigued by the sheer number of alerts [30, 32]. Other studies have found that practice list-size was consistently associated with uptake of technology, with larger practices more inclined to use CDSSs [53], and that GP involvement in the creation of the CDSSs led to feelings of ownership of the technology and increased use of the system [17]. Similar to our study, previous work with UK GPs found that limited IT skills, problems in understanding the output of CDSSs, and difficulty communicating the results of the CDSS to patients discouraged use, and that perceived usefulness, performance, trust in the knowledge base, and ease of use were all factors which influenced GPs' intention to use CDSSs [17, 54].

Other research has proposed frameworks for understanding the low uptake of CDSSs. An American study suggested that speed of use, real time delivery, fit to clinical workflow, and ease of usability would all augment use of a CDSS, whereas CDSSs which asked clinicians to stop performing a usual clinical activity (because of lack of evidence of benefit) were often overridden, and CDSSs which were complex, fell out of date, or asked for a lot of additional information, were disparaged [55]. However, this list of “commandments” for CDSS development was developed with an American clinical workforce in mind and therefore may not translate well to UK clinical practice. Greenhalgh et al.’s Non-Adoption, Abandonment, Scale-up, Spread and Sustainability (NASSS) framework may be relevant for the UK context, and suggests that only when the CDSS tackles a “simple” condition, has excellent usability, with little need for training or troubleshooting, and generates value for clinicians or patients, will it be adopted and scaled up in practice [18]. This maps on generally to our findings which suggested that non-intrusiveness, an actionable clinical outcome and little need for training were all features which facilitated adoption of a CDSS.

### Strengths and limitations

Previous research has looked at likely reasons for clinicians’ poor engagement with CDSSs, but this is one of the first studies that has aimed to identify features of CDSS in UK general practice in order to inform the future development of these tools to ensure uptake and fitness for purpose. However, this is a small qualitative study, and thus provides recommendations which can be further explored in more structured quantitative work, rather than definitive results. One of the limitations of this study was the sampling strategy, which was to approach GPs who were working within two UK medical school networks, rather than identifying GPs practicing across a more representative geographical spread. This resulted in the sampling being mainly part-time GPs, many of whom had an interest in medical education and were in younger age groups (30–39). Younger doctors may be more accepting of automated CDSS than older practitioners. A wider sample of older or full time GPs with no links to medical schools may have given different opinions, and we cannot declare our results reached saturation of information from the full range of GPs, although saturation of themes within our particular sampling strategy was reached. Of note, interviewing, coding of transcripts and theme refinement were carried out by different team members. This has both advantages and disadvantages. It may allow the analysis to be more data-led than a study conducted by a single researcher, where bias may be more likely to occur, but a single researcher who brings knowledge or expertise in the area can achieve deeper understanding of the data by seeing the process through from beginning to end.

### Implications for future CDSS development

This rich exploration of GPs’ views on features of CDSSs that enhance their usability and trustworthiness, allows us to make tentative suggestions for considerations during CDSS development in the future. Importantly, these recommendations address both intrinsic and technical features of individual tools, and the wider clinical context in which multiple CDSSs, multiple demands on GPs and multiple personal issues and morbidities must be better accounted for in design. Individual CDSSs must be developed with a systems approach (taking into account the whole context) rather than just a product approach which focuses only on the development of the specific product without relating to the bigger picture and the influence of the product on other linked issues (e.g., other systems, context of use, and other stakeholders).

We recommend that developers of new prediction, risk-profiling or prescribing safety tools for use in UK primary care patient record systems consider all the domains shown in Table 2 alongside corresponding recommendations.

**Table 2:** Recommendations for further investigation

Domain	Recommendation
Provenance and Transparency	Supply the CDSS alongside an accessible and signposted evidence-base
Threat to autonomy and skills	Include GPs in the development of the tool so it is aligned to their clinical needs and workflow and so that they can recommend it to their peers
Clear communication and management guidance	For any prediction given by the tool, provide an evidence-based management or care guideline for the clinician to follow
	Supply visual communication aids, co-designed with patients, so that clinicians can communicate the outcome of the tool with their patients easily
Sensitivity to wider context	Only release a new tool widely when validation of accuracy, and study of unintended consequences, has occurred in real world settings
	Consider and evaluate contextual effects on accuracy such as age, frailty, and multi-morbidity
	Ensure balance of false positives to false negatives given by tool is appropriate for resource use and does not result in excess harm
User control and flexibility	Allow GP to maintain control, override or dismiss a tool
	Ensure there is a provision to record wider context rationale for over-riding tool use
	Integrate tool appropriately with EHR and ensure self-population from previously recorded data as much as possible
Intrusiveness	Consult with GPs on appropriate balance between having a self-generating pop-up or having a template which can be called up by the GP
Alert proliferation and fatigue	Consider the new tool in the context of all other tools within the system. Is this one really adding value?
	Consider developing technologies which manage multiple tools or prioritise the most important alerts and suppress the rest
	Consider a learning system which learns from the behaviour of GPs and accumulated evidence of the effectiveness of the different tools and alerts to adapt tools’ behaviour
Training and support	Label and signpost online training available for using the tool within the tool itself, e.g. a video showing how it works and how to get the most from it and personalise settings where appropriate
	Sustainability: How expensive and adequate is the support for the tool and will it still be provided over time?

## Conclusions

CDSSs that increase evidence-based decision making in healthcare have the potential to improve quality of care and patient health and outcomes, but will only be of benefit if used widely in clinical environments. Many tools currently available in UK general practice record systems have been conceived and designed with scant regard for usability principles and clinician input, and, as a result of this, are deemed not fit-for-purpose by the users and do not fit with clinician workflow or multi-disciplinary working (such as between primary care and clinical pharmacy). US-based research suggests alerts may even contribute to GP burnout rates [56]. An overemphasis on individual CDSSs design appears to pay scant attention to both the patient context and the clinical context, which includes the existence of multiple CDSSs which at times ‘compete’ for clinician attention. This can result in a proliferation of alerts resulting in none of the tools having their desired impact. We have described a set of recommendations which, if used when each new tool is developed, could rapidly increase the usability and real-world effectiveness of these tools. It is clear from this work that clinician involvement in individual tool design is vital, and that as more and more tools are developed, methods for prioritising the most important ones in any consultation may also be needed, to reduce alert fatigue. Individual design must take account of both consultation context and dynamics but also the broader technological context by adopting a systems approach to design. We hope that further development and potential adoption of these suggested recommendations will increase the scrutiny and quality of new CDSSs coming to GP electronic health record systems and will contribute to enhanced patient care in the age of digital health.

## Supplementary Information

**Additional file 1.** Interview Topic Guide .

## Data Availability

The transcripts of interviews generated and analysed during the current study are not publicly available because individual privacy could be compromised, but are available from the corresponding author on reasonable request.

## Abbreviations

AI: Artificial intelligence
BSMS: Brighton and Sussex Medical School
CDSS(s): Clinical decision support system(s)
COREQ: Consolidated criteria for reporting qualitative research
EHRs: Electronic health records
GP: General practice or general practitioner
HBA1c: Glycated haemoglobin
NASSS: Nonadoption, abandonment, scale-up, spread, and sustainability framework
QOF: Quality and outcomes framework
UK: United Kingdom
