# Supplementary material for: Barriers and facilitators to the adoption of electronic clinical decision support systems: a qualitative interview study with UK general practitioners
Source: BMC Med Inform Decis Mak. 2021 Jun 21;21:193. doi: 10.1186/s12911-021-01557-z (PMC8215812; doi:10.1186/s12911-021-01557-z)
Supplement: Supplementary file 1 — Additional file 1. Interview Topic Guide . [file 12911_2021_1557_MOESM1_ESM.pdf]

Appendix 1: Topic Guide for GPs

Preamble:

At BSMS we have been developing a predictive algorithm to identify people with dementia who might not yet have been recognised as such by their GP. This would ideally run within GP patient software and alert GPs when one of their patients is likely to be developing dementia.

This algorithm has been developed using data from a large database of GP patient records.

Although this is just a prototype, it is one example of how data from patient records can be used to create computer systems providing advice, recommendations or alerts which run in GP patient records software (EMIS, systmOne etc).

Question 1:

Can you tell me about your experience of these types of alerts, risk scores, prescribing advice, or other clinical decision aids which are enabled within your patient records software?

Prompts: what do you think of them, do you use them, which ones do you use, which do you not use and why, what do you like about it, what do you dislike?

Question 2:

Are there any particular features or functions that you like in these types of computer systems providing advice, recommendations or alerts? If so, can you describe these? Are there any particular features you really don't like? What are these?

Question 3:

How good would a computer system providing advice, recommendations or alerts need to be for you to be willing to use it regularly? Would you prefer that it found every possible case (sensitivity) or prefer to avoid false positives (specificity)?

Question 4:

What is the impact of computer systems providing advice/recommendations or alerts on your interactions with patients? How easy do you find it communicating with patients about a suggestion given by an alert or algorithm? What do you do if there is a difference between your opinion and the decision given by the computer system?

Question 5:

What are the possible unintended consequences of bringing computerised decision support (or AI) into the general practice clinic?

Question 6:

Do you identify any ethical or professional/clinical issues with the increasing implementation of computer systems providing advice/recommendations or alerts in general practice? What are these?

Question 7:

Do you have any suggestions about how GPs could be better involved in the design and implementation of computer systems providing advice, recommendations or alerts?

Question 8:

Computer systems providing advice, recommendations or alerts are a kind of artificial intelligence. What concerns do you have about "artificial intelligence" (AI) in general practice becoming more widespread? Do you see this as a good thing or a bad thing? How could your concerns be addressed to make you feel confident that development of AI will be for patient benefit?
